# Loss of correction in unstable comminuted distal radius fractures with external fixation and bone grafting -a long term followup study

**DOI:** 10.1186/1749-799X-6-23

**Published:** 2011-05-21

**Authors:** PuttaKempa Raju, Sunil Gurpur Kini

**Affiliations:** 1 Victoria Hospital, Bangalore Medical College and Research Institute, Bangalore, India; 2 Tan Tock Seng Hospital, Singapore

## Abstract

Over the years, management of complex distal radius fractures by closed means has often failed leading to late collapse. We have chosen the principle of ligamentotaxis using external fixation and bone grafting in this study to prevent late complications. Eighty one patients with complex distal radius fractures belonging to Type IV A, IV B, IV C of Universal classification were treated with an AO external fixator between 1995 and 2001. Mean age group was 38. 47 years with longest follow up of 7 years. Bone grafting was done primarily in 20 patients and early grafting (within 3 weeks) in 5 patients. Statistically significant differences were observed between the two groups(with or without bone grafting) with respect to postoperative values of (radial length, radial tilt and volar tilt). Results were assessed based on Sarmientos criteria. 56 patients had excellent results, 9 had good results and 16 had poor results. Late collapse with decreased radial length was observed in 18 patients who did not undergo bone grafting. Mean grip strength was 63 percent. Osteoarthritic changes were noted in 20 patients. We conclude that accurate anatomic reduction is necessary for achieving good to excellent functional and cosmetic results. Bone grafting is the mainstay of treatment in comminuted distal radius fractures along with fracture stabilisation.

## Introduction

The management of complex distal radius fractures is controversial. The approach to these fractures range from cast immobilisation, external fixation, plating techniques including fragment specific fixation. The need for external fixation of these fractures to obtain accurate anatomic reduction has increased the interest in these devices. Late collapse of these fractures has been a subject of debate for last decades. Bone grafting for large metaphyseal voids is well described entity to avoid collapse and morbidity. Open placement of pins has led to fewer complications including avoidance to injury to superficial radial nerve and less damage to tendons and soft tissues. Malunion and late deformity even after external fixation has been reported. Proper selection of patients for external fixation and timely bone grafting has resulted in best possible functional and cosmetic results. We present a long term followup of complex distal radius fractures treated by external fixation and discuss the results in terms of restoration of anatomy and function.

## Materials and methods

The study conducted at Victoria hospital attached to Bangalore Medical College and Research Institute comprised of 70 men and 11 women and the mean age was 38. 47 years. 57 of them were involved in heavy labour,3 were students and rest were involved in office work. The mechanism of injury was motor vehicle accident in 69 patients, fall from a height in 12 patients. 46 of them involved the dominant hand and 35 the other. 4 of them were open fractures-2 grade I,1 grade II and 1 grade IIIA.

Indications-Selection criteria included

1. A3, C2&C3-AO Classification

2. Frykmans V-VIII Fractures

1. Loss of position following closed reduction

3. Bilateral Colles'

4. Compound comminuted fractures

5. Unstable Fractures-

a) > 2 mm spread of fragments

b) Intra-articular extension with comminution

c) > 10 deg. angulation

d) Ulnar neck fracture

e) Shortening > 10 mm.

Assessment-On arrival to the hospital a detailed history was elicited and associated injuries were documented. Standardised Anteroposterior and lateral radiographs were taken and after thorough evaluation, unstable comminuted fractures were enrolled into the study. Majority of patients treated by external fixation were of closed comminuted (85. 7%) and intraarticular(100%) in nature. 67 patients reported to us immediately and 14 within 48 hours of trauma. External fixator was applied using the Shanz principle of ligamentotaxis. 77 of them were operated immediately and 4 of them were operated on days 2,3, 5 and 7 (loss of initial reduction).

Technique- With the patient in supine position under anaesthesia and tourniquet, the prepared extremity is draped. Four pins were used 2 in the radius and 2 for the second metacarpal. The proximal most pin for the radius and the distal most pin in the metacarpal is introduced first after predrilling. Open pin placement avoids damage to the intrinsic muscles and tendons and avoids eccentric pin placement. After precise pin application the fixator bar is applied, fracture fragments reduced by palmar translation of the fragments with the wrist in neutral position, distraction was applied and fixator clamps tightened. Reduction was verified under C arm and then the rest two pins inserted. Post operatively antibiotics were used for 2 doses in closed and 3 days in open fractures.

Mobilisation of finger at Metacarpophalangeal and Interphalangeal joints, forearm supination and pronation movements and shoulder exercises were started from the day after surgery. Regular followup visits were carried every week. External fixator was removed after confirming radiological union at a mean of 8 weeks and vigorous physiotherapy started.

Bone grafting was done in 25 patients via dorsal approach in the following situations.

• Osteoporotic bone

• Metaphyseal voids

• High energy trauma in younger individuals

• Large gaps due to absorption of metaphyseal fragments.

Bone graft was harvested from the iliac crest through a limited approach. The grafts were packed tightly through the fracture site. Anatomic reduction was obtained by minimizing the articular incongruity to less than 1 mm.

## Results

The followup duration ranged from 38 months to 91 months with a mean of 62 months. The functional evaluation of end results was done by the Gartland and Werley's point system and anatomical results by Sarmiento's criteria.

The followup examination included detailed questionnaire based on demerits system to assess the results. All the patients were evaluated by the senior author. Statistical analysis of data was done using Pearson coefficient or chi square test. Grip strength was assessed using using a dynamometer. The contralateral limb was used as control. Reduced grip strength was noticed inpatients who had increased dorsal tilt and decreased radial length of more than 10 mm. 56 patients had excellent results,9 good and 16 poor results. Late collapse was noticed in 22 patients who did not undergo bone grafting. The same was observed in osteoarthritic changes secondary to late collapse. The presence of post traumatic arthritis in 20 patients were graded as per the table (Table 1)

**Table 1:** Grading of Radiocarpal arthritis

Grade	Arthritis
0	No changes
I	Minimal narrowing of joint space-medial/lateral in the Radiocaroal joint
II	Marked narrowing of joint space, osteophyte formation
III	Severe narrowing of joint space, osteophyte formation, cyst formation

Arthritis in Radiocarpal joints developed in 20 of 56 patients without bone grafting and none in the grafted group (25 cases). In all these the articular incongruity was more than 6 mm. Arthritis limited to distal radioulnar joint was seen in 3 patients. The occurrence of die punch fragment adversely affected the late results. Die punch fragments were reduced anatomically in all the patients except in three patients and all of them developed residual radiocarpal incongruity. Although the residual articular incongruity frequently resulted in posttraumatic arthritis, the minimal stepoff in severely comminuted fractures in which the anatomical reduction and congruity was restored and maintained led to significantly better overall results (Table 2).

**Table 2:** Articluar Incongruity grading

Grading	Step off (in mm)
0	0-1
1	1-2
2	2-3
3	>3

**Figure 1:**
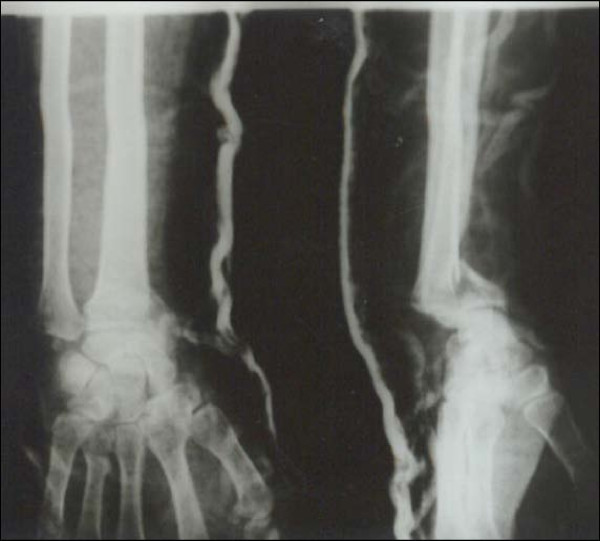
**Case 1 Preoperative Radiograph - Anteroposterior and Lateral view**.

**Figure 2:**
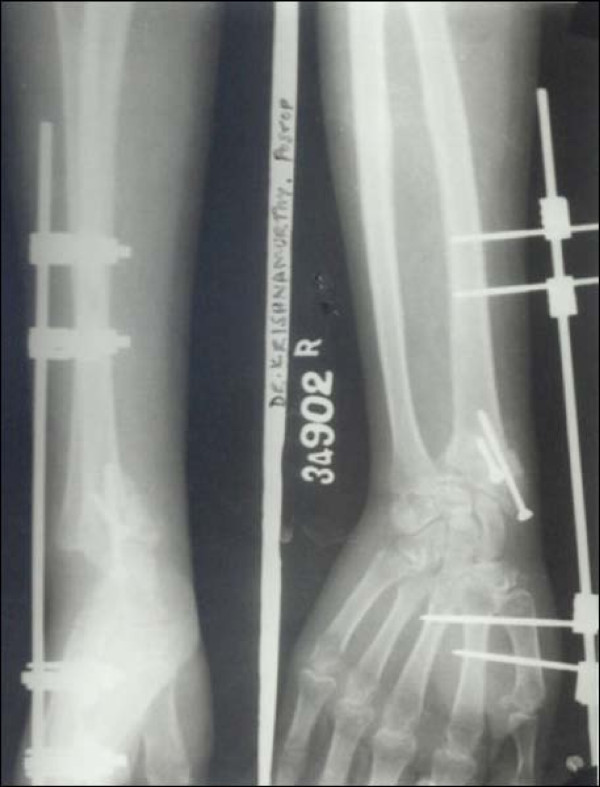
**Case 1 Postoperative Radiograph - Anteroposterior and Lateral view**.

**Figure 3:**
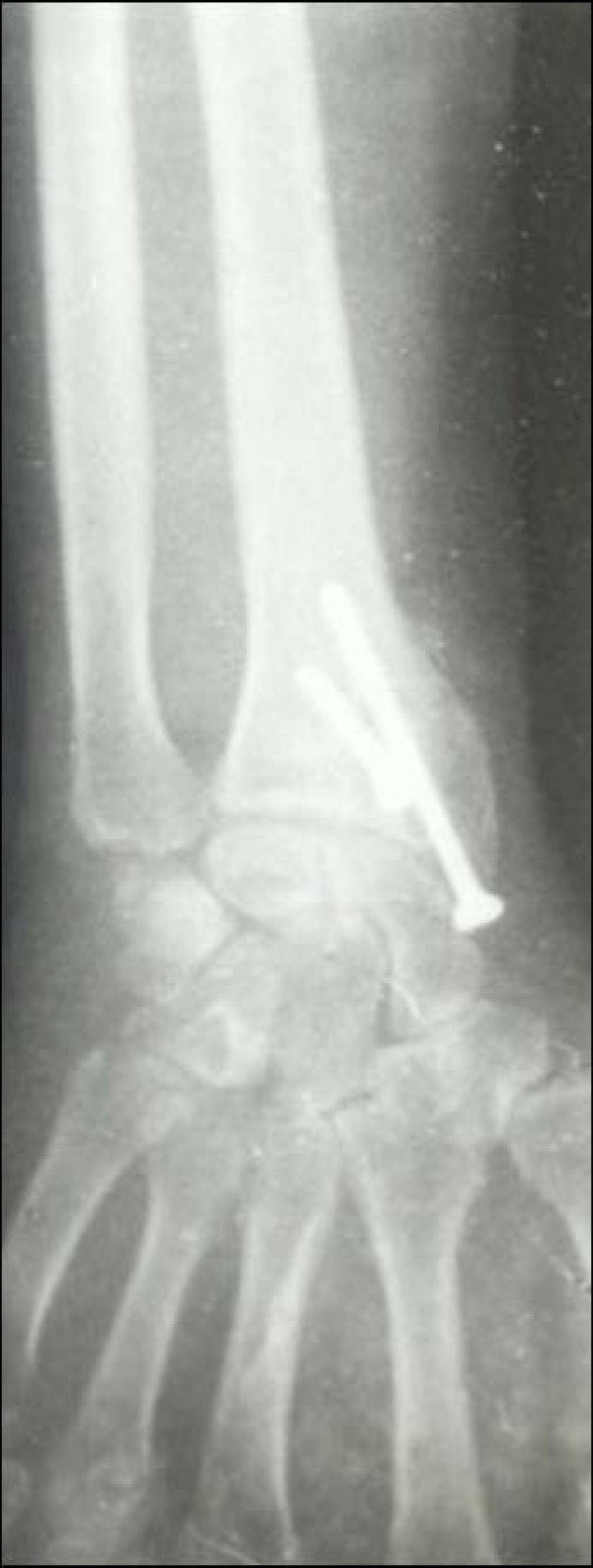
**Case 1 Radiograph (Anteroposterior view) at 7 year followup**.

**Figure 4:**
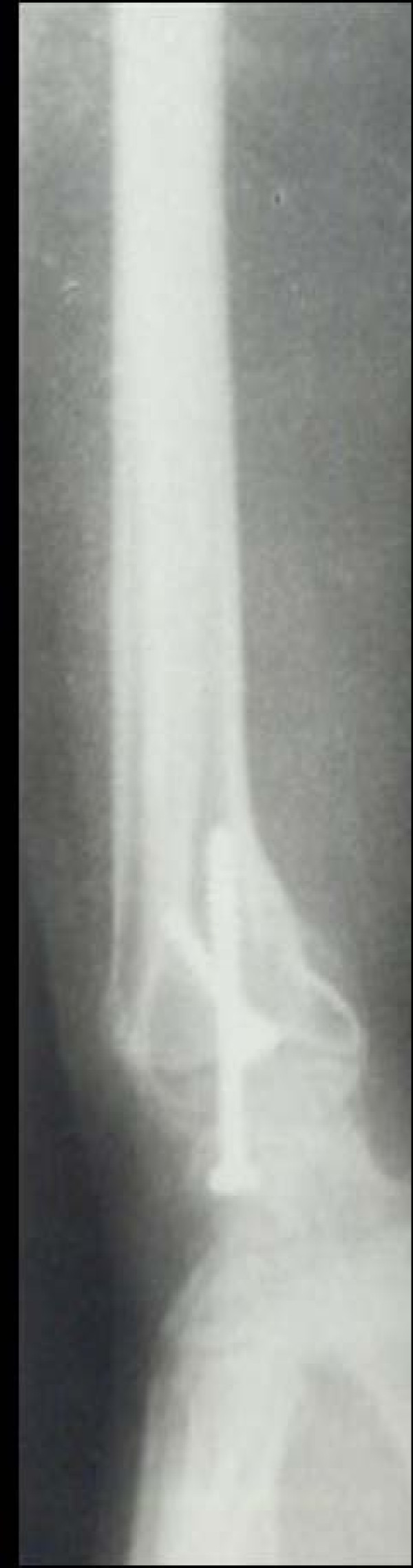
**Case 1 Radiograph (Lateral view) at 7 year followup**.

Case 1 - Figure 1, Figure 2, Figure 3, Figure 4

**Figure 5:**
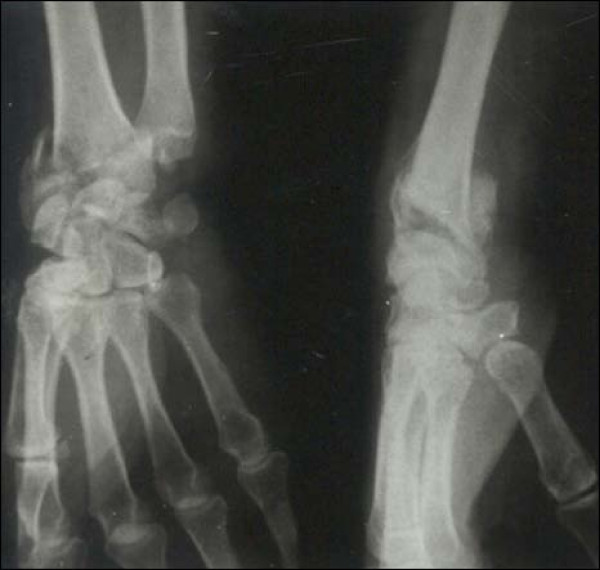
**Case 2 Preoperative Radiograph Anteroposterior and Lateral view**.

**Figure 6:**
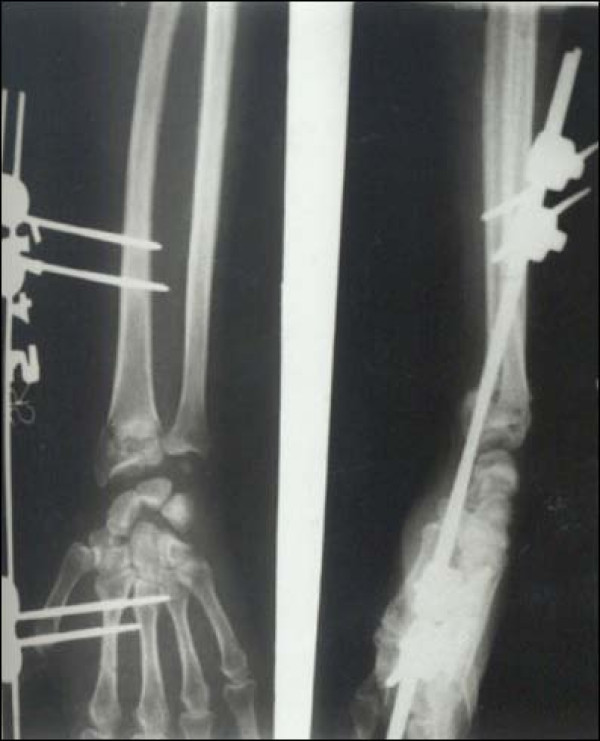
**Case 2 Immediate Postoperative Radiograph Anteroposterior and Lateral view**.

**Figure 7:**
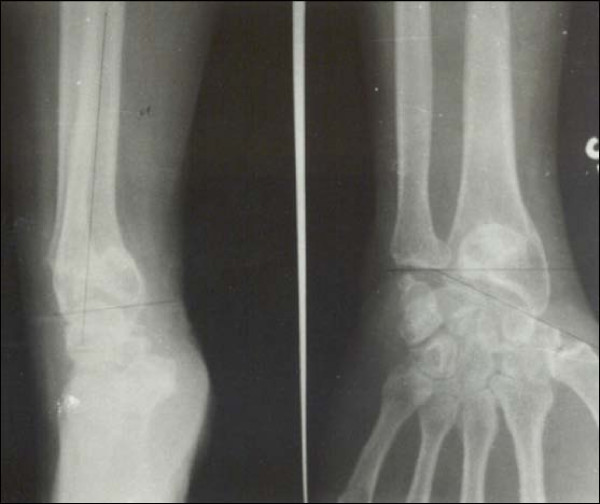
**Case 2 Radiograph at 7 year followup**. Anteroposterior and Lateral view.

Case 2 - Figure 5, Figure 6, Figure 7

**Figure 8:**
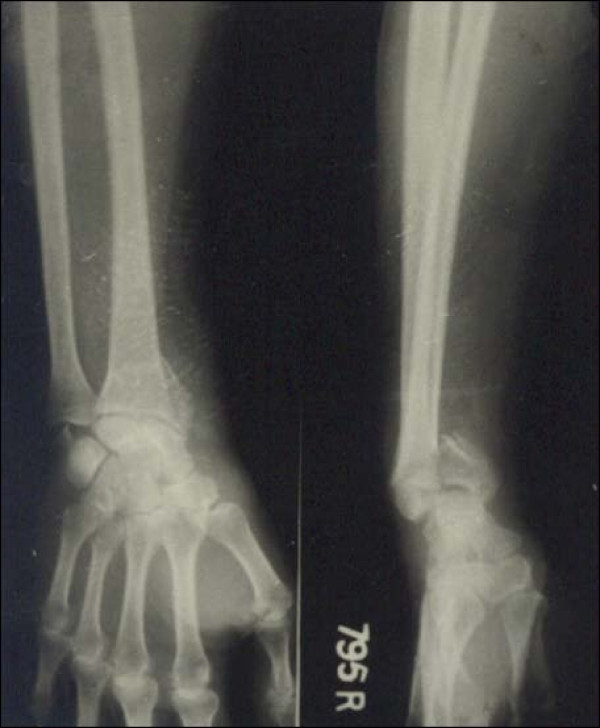
**Case 3 Preoperative Radiograph Anteropsterior and Lateral view**.

**Figure 9:**
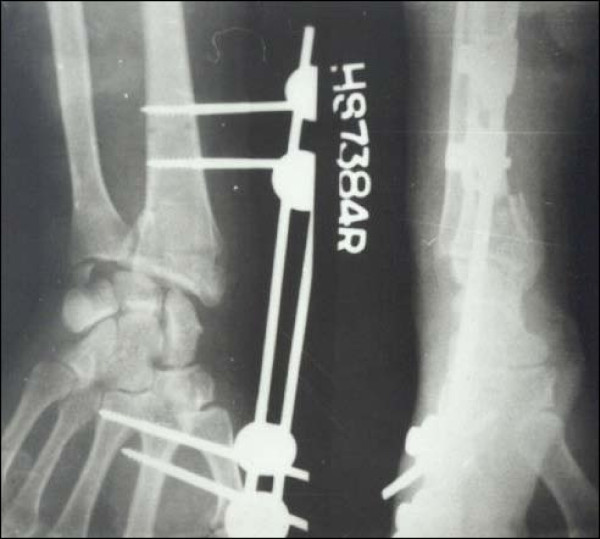
**Case3 Postoperative Radiograph (Anteroposterior and Lateral view)**.

**Figure 10:**
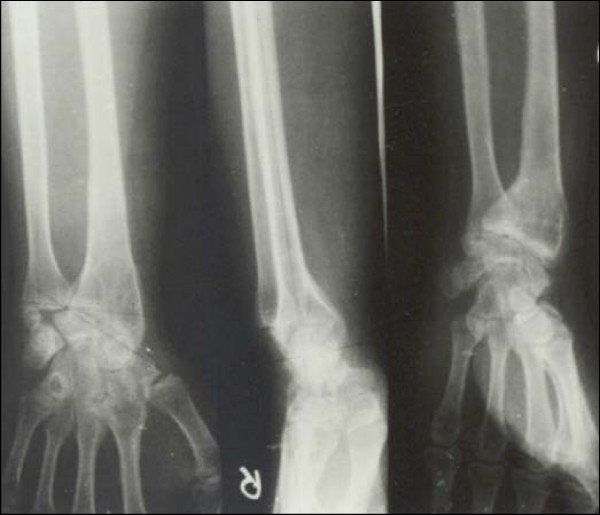
**Case 3 Radiograph at 7 year followup Anteroposetrior and Lateral view**.

Case 3 - Figure 8, Figure 9, Figure 10.

## Discussion

80 per cent of axial loads at the wrist are supported by the distal end of the radius and 20 percent by the triangular fibrocartilage and the distal end of the ulna[1]. The limitation of external fixation to achieve articular congruity in the comminuted intra-articular fractures of the distal radius has been documented previously[2]. This could be because external fixation alone does not expand crushed cancellous bone and cannot work without soft tissue hinges [3].

Skeletal traction maintained by a half frame external fixator between the radius and second metacarpal bone appears to provide appropriate stabilization of the fragments. External fixator provides stability and fixed traction, prevents shortening due to either bone loss or late resorption of cancellous bone from the metaphysis. A study by Sommerkamp et. al stated that the loss of four millimeters of radial length in the dynamic-fixator group over the course of treatment was significantly greater than the one-millimeter loss in the static-fixator group [4].

Current concepts reflect the growing popularity of external fixation of complex distal radius fractures because it provides easy accessibility of wound care and it can be combined with secondary procedures like bone grafting and skin coverage.

### Age and sex incidence

Age group ranged from 20 yrs to 58 yrs with mean of 38. 47 yrs. Increased incidence of these fractures in males(88. 46%) in our series (Cooneyet. al 11. 6%) especially in adults is attributed to high level of activity in males and road traffic accidents(84. 61%) among riders of two wheelers.

### Type of fracture/Classification

Majority of fractures were closed(94. 23%). All fractures were of Type IV Universal classification 2 Type IV A, 31 Type IV B, 19Type IV C.

### Timing of fixator removal

Recommended duration of external fixation use varies and sometimes extends to between 8 and 12 weeks [5]. Until fixator removal, patients were followed up once a week. Average duration for removal was 8 weeks indicated by radiological fracture union which was the main criteria. In literature the duration of use of fixator ranges from 8 to 12 weeks. Fair and Poor functional results can be attributed to extended period of application of external fixation in 4 patients.

### Bone grafting

Bone grafting is the mainstay of treatment in any distal radius fractures with large metaphyseal void which are prone to collapse at later date. Cancellous bone grafts harvested from the iliac bone reduced the period of external fixation and supported the articular surface. Packing cancellous bone chips into these comminuted fractures increased the rigidity of reduction fourfold [6]. Bone grafting was carried out either primarily or secondarily and also on the acceptability of patients for bone grafting. Bone grafting improved the anatomical alignment of the fragments and the articular congruity and allowed early mobilization of the wrist and fingers. The external fixation does not provide the absolute stability to maintain the comminuted intrarticular fractures. It takes a longer time for the fracture gap to be filled by new bone formation. In study of Overggard et. al over a seven-year follow-up, seventeen (30 per cent) of their fifty-six patients had radiographic evidence of osteophytes and eight patients (14 per cent) had advanced radiographic changes [7]. In our study late collapse was not noticed in patients who underwent bone grafting after 7 year followups. By pushing the grafts towards the distal articular surface many of the die punch fragments which cannot be reduced by ligamentotaxis alone can be adequately lifted, reduced and supported to achieve congruent articular surface [8]. Bone grafts supply an interosseous distension force which enhances the ligamentotaxis and helps to line up the juxtaarticular bone fragments to maintain the integrity of the distal radius.

Combination of ligamentotaxis and cancellous bone grafting produced excellent clinical and radiological results. As Green pointed out good functional results usually follow good anatomical results. Our method utilized both biological and mechanical effects of bone grafting enabling us to reduce the duration of external fixation and to obtain internal trabecular healing. Tight packing with bone grafts produces better load bearing, fills space and stretches and tightens the residual periosteum. Then compressive strength of bone tissue is proportional to the square of the apparent density. So highly compact cancellous bone grafts provides good stability in metaphyseal fractures.

### Anatomical results

We believe that it is the quality of reduction that determines the clinical outcome. Thus the aim of external fixation is to obtain and maintain an accurate anatomic reduction of the fracture fragments and to prevent collapse, malunion, deformity and late osteoarthritis. Maintenance of radial length results in good functional outcome. The average radial height in AP view is 11 to 14 mm and a height of less than 4 mm corresponds to poor Haddad et. al in his study of 43 patients showed that all but two of the patients (5%) had a volar tilt of up to 16°, the radial length was restored in 77% and excessively shortened by 3-4 mm in 9 patients (23%) [9]. Leung et. al in his series showed loss of radial articular angle (mean 2. 2 degrees) after removal of the external fixator[10]. In our series in 95% of cases, radial length of more than 6 mm was maintained. Decrease in the excellent functional results with respect to the maintenance of radial length of more than 6 mm is due to the non cooperation of patients for physiotherapy and longer periods of immobilisation. In our series restoration of the normal volar tilt in 90% of cases resulted in excellent anatomical result. The excessive dorsal tilt produces a dinner fork deformity and decreases the range of palmar flexion and also causes midcarpal instability due to changes in load distribution. The collapse of the articular surface was not encountered in the dorsal angulations in our series as the patients were not allowed to perform extension for an additional 2 weeks after external fixator removal.

### Range of movements

Most patients regained good range of motion of wrist and forearm all obtained normal finger movements. Inspite of satisfactory reduction in 2 cases, persistent wrist stiffness was encountered. This limitation of joint motion is well tolerated by patients as the majority of hand tasks can be accomplished with 70% of maximal range of wrist movements which is revealed in this study. For routine functional activities we require 35 degree of dorsiflexion and 10 degree of palmar flexion of the wrist has been achieved in all the cases in the study. No other method appears to be technically simple and to give such excellent results.

### Demerits

Posteromedial fragments, Indirect control of fragments, No accurate reduction of intraarticular fragments, Excessive distraction. Reduction and maintenance of reduction is more difficult using bridging external fixation because there is indirect control of the distal fragment, which depends on ligamentotaxis; this may not be successful in restoring the volar tilt or the radial length [11]. Difficulty in achieving volar tilt also may be due to the fact that the stout palmar radiocarpal ligaments reach maximum length before the z-shape dorsal ligaments, preventing the latter from pulling the dorsal aspect of the distal end of the radius into its normal palmar inclination[12]. Considerable transfer of load onto the ulna occurs with progressive dorsal angulation of the distal end of the radius [13].

### Complications

Tissue perforation (n = 2), Pin tract infection (n = 2), Pin loosening (n = 3), bending and breaking of pins(n = 3), Loss of reduction(n = 3), Stress fractures(n = 2), Inflammatory reactions(n = 4), Osteolysis of cortices(n = 3), Spontaneous pullout of pins(n = 2), Neuroma of sensory branch of radial nerve(n = 2), Reflex sympathetic dystrophy (n = 3), Wrist stiffness (n = 3), Rupture of EPL tendon(n = 1), Osteoarthritis of wrist(n = 2).

Loss of reduction was seen in 3 patients only that confirms to previous studies, which show that external fixation effectively maintains the reduced position [14].

Restriction of movements at the wrist has been attributed to the extended period of application of external fixation and improper physiotherapy and also due to associated injuries in the ipsilateral limb which interfered with the physiotherapy. Use of open pin insertion technique with a predrilled system has reduced the injury to both tendon and nerves. Radial sensor nerve is generally not at risk when pins are placed 10 cm proximal to the radial styloid process by this technique. Open pin placement and pin insertion with sharp drill bits and improved fixation with better thread design and insertion of pins at an angle of 60 degrees to each other increased the purchase of pins in the bone and decreased complications like pin bending, loosening and breakage. Papadonikolakis et. al in their study concluded that more than 5 mm of wrist istraction increases the load required for the flexor digitorum superficialis to generate MCP joint flexion for the middle, ring, and small fingers. For the index finger, however, as much as 2 mm of wrist distraction significantly increases the load required for flexion at the MCP joint[15].

Complex distal radius fractures pose a significant challenge to the practicing surgeon because of the inherent tendency to collapse resulting in malunion, deformity. loss of function and late osteoarthritis. Fair and poor results were attributed to associated injuries and extended period of application of external fixator. Lunate fragments which could not be reduced by external fixation required open reduction, fixation with K wires and bone grafting. Ulnar styloid process fractures were not actively treated in this study. Late collapse of the articular surface led to early arthritis. Bone grafting should be performed to obtain good articular congruity and to prevent deformity. Although AO external fixator provides absolute rigidity and stability, restoration of original palmar tilt could not be achieved in all cases despite maintaining radial length and radial. The restoration of palmar tilt requires multiplanar ligamentotaxis or a pin in the dorsal fragment. Majority regained more than 63 percent of grip strength. It is decreased in patients with increased radial tilt, associated injuries and prolonged immobilisation. The final outcome of functional results in complex distal radius fractures depends on patient selection, fracture morphology, obtaining accurate reduction and maintaining it by external or internal fixation, bone grafting inpatients with large metaphyseal void, patients compliance towards physiotherapy and associated injuries.

## Competing interests

The authors declare that they have no competing interests.

## Authors' contributions

PKR was the main operating surgeon in all cases and follow up of the cases. GSK was involved in maintaining the records of the patients, review of literature and writing up the paper. All authors read and approved the final manuscript.

## Author's Information

PuttaKempa Raju M. B. B. S, D. (Orth), M. S(Orth)

Department of Orthopaedics, Bangalore Medical College and Research Institute, Bangalore, India

Sunil Gurpur Kini M. B. B. S, M. S(Orth), D. N. B(Orth), M. Ch(Orth), M. R. C. S(Glasg),

M. R. C. S(Edin), MNAMS, Dip. SICOT Tan Tock Seng Hospital, Singapore
